# Snapshot into the Type-2-Diabetes-Associated Microbiome of a Romanian Cohort

**DOI:** 10.3390/ijms232315023

**Published:** 2022-11-30

**Authors:** Gratiela Gradisteanu Pircalabioru, Mariana-Carmen Chifiriuc, Ariana Picu, Laura Madalina Petcu, Maria Trandafir, Octavian Savu

**Affiliations:** 1 Research Institute of University of Bucharest (ICUB), 050095 Bucharest, Romania; carmen.chifiriuc@bio.unibuc.ro; 2 Academy of Romanian Scientists, 050045 Bucharest, Romania; 3 Romanian Academy, 010071 Bucharest, Romania; 4 Faculty of Biology, University of Bucharest, 050095 Bucharest, Romania; 5 “N.C. Paulescu” National Institute of Diabetes, Nutrition and Metabolic Diseases, 020042 Bucharest, Romania; arianapicu@gmail.com (A.P.); madi_petcu@yahoo.com (L.M.P.); octavian.savu@umfcd.ro (O.S.); 6 Department of Doctoral School, “Carol Davila” University of Medicine and Pharmacy, 5th District, 050474 Bucharest, Romania; maria.f.trandafir@gmail.com

**Keywords:** microbiota, microbiome, diabetes, metabolites, hypertension, metabolic disease

## Abstract

The prevalence of type 2 diabetes mellitus (T2D) is alarmingly increasing worldwide, urgently calling for a better understanding of the underlying mechanisms in order to step up prevention and improve therapeutic approaches. It is becoming evident that the gut microbiota seem to have an endless capacity to impact T2D. In this study, we profile the gut microbiome patterns in T2D patients from Romania, by using quantitative Real-Time PCR and next generation sequencing. We enrolled a total of 150 individuals (105 T2D patients, 50 of them without metformin treatment and 45 healthy volunteers). The levels of potentially beneficial butyrate-producing bacteria were significantly reduced, while potentially pathogenic microorganisms such as *Enterobacteriaceae* and *Fusobacterium* were enriched in T2D patients. We evaluated the correlation between clinical parameters and gut microbiota and identified the genera Bacteroides, *Alistipes*, *Dialister*, *Bilophila* and *Sutterella* as possible detrimental factors in T2D. Our findings suggest that the gut microbiota may be a potential target in novel approaches to halt the development of T2D-associated complications.

## 1. Introduction

With a worldwide prevalence projected to reach over 700 million patients by 2045 [1], type 2 diabetes (T2D) is a chronic metabolic disease marked by hyperglycemia and linked with insulin resistance and/or insufficient production of pancreatic insulin. The PREDATORR study was the latest national study to systematically assess the prevalence of DM in the Romanian adult population [2]. The study showed a T2D prevalence of 11.6%. The prevalence of T2D was higher in men than women and in the elderly [2]. Given its rising burden and its association with many micro- and macrovascular complications leading to renal failure, hypertension, limb amputations, blindness and susceptibility to infections, T2D is a top health priority for the World Health Organization (WHO) and the United Nations (UN) [3,4].

Among the players involved in T2D pathophysiology, the gut microbiota may significantly contribute to disease development by impacting the host glucose metabolism pathways [5].

So far, different microbiome alterations have been described in the case of T2D patients. T2D subjects were shown to harbor a high proportion of Proteobacteria and Bacteroidetes and a lower relative abundance of Firmicutes [6]. Other studies have reported a significant increase in Firmicutes and Proteobacteria and a reduction in Bacteroidetes leading to an enhanced Firmicutes/Bacteroidetes(F/B) ratio in T2D compared to non-diabetic subjects [7,8]. Moreover, an enrichment of opportunistic pathogens is frequently reported in the T2D microbiome including the species *Escherichia coli, Bacteroides caccae*, *Clostridium ramosum*, *Eggerthella lenta*, *Clostridium hathewayi* and *Clostridium symbiosum* [9,10,11]. 

Microbial metabolites are an important factor shaping host health. Short chain fatty acids (SCFAs) impact glucose metabolism via the coupling action with selected G-protein-coupled receptors (GPRs). For instance, GPR119 and GPR43 stimulation promotes incretin GLP-1 secretion by enteroendocrine L-cells [11,12,13,14]. GLP-1 protects β-cells from apoptosis, stimulates glucose-induced insulin release from β-cells and suppresses glucagon secretion [15]. Acetate, the most abundant SCFA, is absorbed by the gut epithelium, and after transport to the liver is distributed to peripheral tissues [16]. 

Another important SCFA is butyrate, the main substrate and energy source for colonocytes and of paramount importance for host gut homeostasis, exhibiting anti-inflammatory properties [17,18,19,20]. T2D patients are consistently being reported to be low in butyrate-producing microbes including *Faecalibacterium prausnitzii*, *Subdoligranulum*, *Roseburia intestinalis*, *Roseburia inulinivorans*, *Ruminococcus* and *Eubacterium rectale* [7,8,9,11,21].

T2D has also been linked with a significant reduction in *Akkermansia muciniphila*, a microbe which plays a crucial role in maintaining the integrity of the intestinal mucin layer and reducing inflammation [22,23].

In this study, we profile the gut microbiome patterns in Romanian T2D patients. We have enrolled a total of 150 individuals (105 T2D patients and 45 healthy volunteers) and analyzed their microbiota using molecular biology techniques (quantitative Real-Time PCR and next generation sequencing). Finally, we correlated the microbiome profiles with the clinical parameters to identify members of the microbiota associated with poor T2D prognosis.

## 2. Results

Our study enrolled 150 subjects and aimed to explore the microbiota patterns in T2D Romanian patients and to associate the identified microbial signatures with microbially derived metabolites (particularly SCFAs) and clinical parameters.

We initially compared the fecal microbiota of T2D patients (*n* = 105) with that of healthy volunteers using quantitative Real-Time PCR of the 16S rRNA gene. To this end, we used SYBR Green primers recognizing different phyla and bacterial populations and universal primers targeting Eubacteria 16S for normalization.

**Figure 1:**
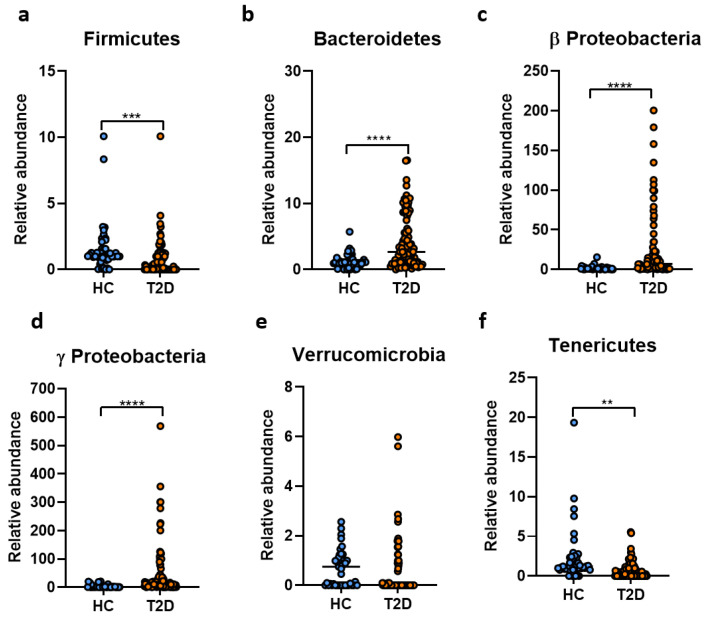
Microbiota analysis in T2D patients (*n* = 105) versus healthy controls (*n* = 45). The relative abundance of the Firmicutes (**a**), Bacteroidetes (**b**), Beta Proteobacteria (**c**), Gamma Proteobacteria (**d**), Verrucomicrobia (**e**) and Tenericutes (**f**) bacteria in fecal samples harvested from healthy individuals and T2D patients. **, *p* < 0.01; ***, *p* < 0.001, **** *p* < 0.0001.

We first investigated the relative abundance of the main phyla which make up the gut microbiota (Figure 1a–f). T2D patients were significantly lower in Firmicutes and higher in Bacteroidetes bacteria when compared to the healthy controls (HC) (Figure 1a,b). Fecal samples collected from T2D patients were highly enriched in *Proteobacteria* families, particularly in Beta Proteobacteria (Figure 1c) and Gamma Proteobacteria (Figure 1d). Even though the HC cohort was higher in Verrucomicrobia, this difference was not statistically significant between the two experimental groups (Figure 1e). Moreover, members of the Tenericutes phylum were more abundant in case of the HC compared to the T2D cohort (Figure 1f).

**Figure 2:**
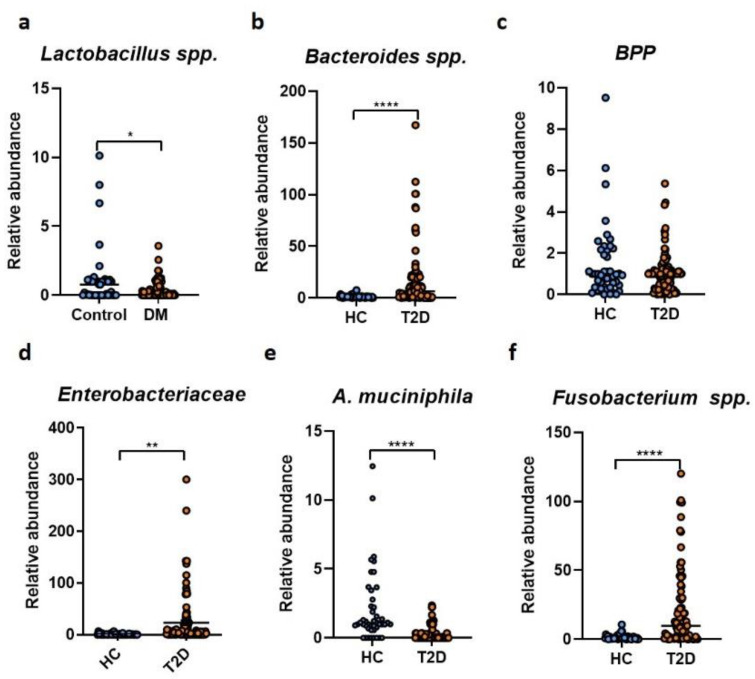
Microbiota analysis in T2D patients (*n* = 105) versus healthy controls (*n* = 45). The relative abundance of the *Lactobacillus spp*. (**a**), *Bacteroides spp*. (**b**), *Bacteroides–Porphyromonas–Prevotella* (**c**), *Enterobacteriaceae* (**d**), *A. muciniphila* (**e**) and *Fusobacterium spp*. (**f**) in fecal samples harvested from healthy individuals and T2D patients. *, *p* < 0.05, **, *p* < 0.01, **** *p* < 0.0001.

We next investigated the abundance of different bacterial populations of the gut microbiome (Figure 2a–f). Lactobacilli species, well known for their potential health benefits, were significantly reduced in the T2D group (Figure 2a). Conversely, T2D patients were significantly enriched for *Bacteroides spp.* (Figure 2b). While no significant differences were found in the case of the *Bacteroides–Prevotella–Porphyromonas group (BPP)* (Figure 2c), the *Enterobacteriaceae* family was positively correlated with T2D (Figure 2d).

*Akkermansia muciniphila*, a gut microbe constantly associated with metabolic health [24], was significantly diminished in the T2D cohort compared to the HC group (Figure 2e). In addition, T2D patients were characterized by a significant increase in *Fusobacterium* species (Figure 2f).

**Figure 3:**
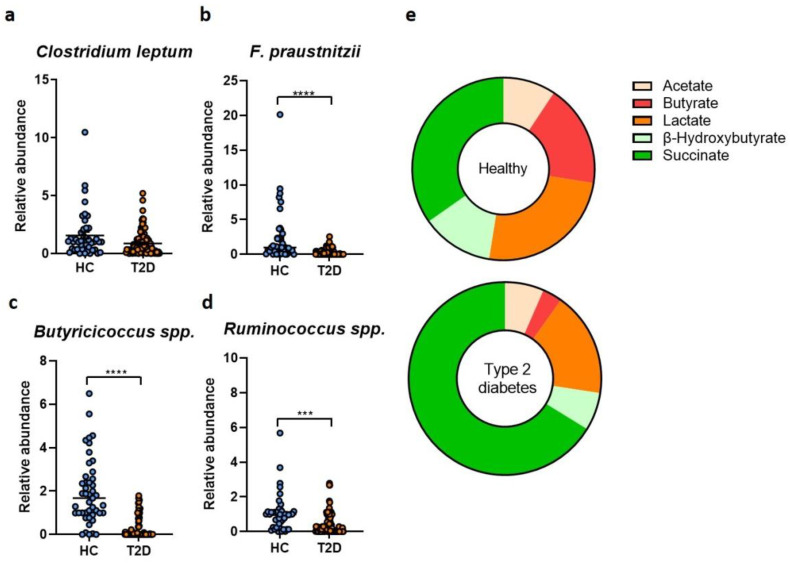
Microbiota and metabolite analysis in T2D patients (*n* = 105) versus healthy controls (*n* = 45). The relative abundance of the *Clostridium leptum* (**a**), *F. prausnitzii* (**b**), *Butyricicoccus* (**c**), *Ruminococcus* (**d**) in fecal samples harvested from healthy individuals and T2D patients; (**e**) metabolites quantification in fecal samples harvested from healthy individuals and T2D patients ***, *p* < 0.001, **** *p* < 0.0001.

Further analysis of the gut microbiota revealed that the T2D microbiome was associated with a significant reduction in butyrate-producing microorganisms including the *Clostridium leptum* group (Figure 3a), *Faecalibacterium prausnitzii* (Figure 3b), *Butyricicoccus* *spp.* (Figure 3c) and *Ruminococcus* (Figure 3d). These microbiome patterns were further correlated with a reduction in butyrate concentration in T2D patients compared to healthy controls (Figure 3e). The fecal metabolome of T2D patients was also characterized by lower acetate and beta hydroxybutyrate and an increase in succinate levels (Figure 3e).

We next analyzed the microbiota patterns in T2D using next generation sequencing. Since anti-diabetic drugs, particularly metformin, exert an important effect on the gut microbiota [20], we selected for sequencing a subset of 50 patients without metformin treatment. The fecal microbiota of the 50 T2D subjects enrolled in the study was evaluated by Ion torrent sequencing. We stratified the analyzed patients based on how likely they are to develop diabetic complications, specifically into newly diagnosed T2D (less than 3 years from the T2D diagnostic) and chronic T2D (more than 3 and up to 10 years of diagnostic). In our cohort, 24 patients were diagnosed with T2D less than 3 years whereas the rest of 26 had chronic T2D. 

The top bacterial OTUs (Operational Taxonomic Unit—used to classify groups of closely related individuals, clusters of organisms, grouped by DNA sequence similarity of a specific taxonomic marker gene) in diabetic patients were calculated by taxonomic analysis. The top 30 OTUs identified in the microbiota of diabetic patients included *Bacteroidaceae*, *Bacteroides*, *Sutterellaceae*, *Enterobacteriaceae*, *Sutterella*, *Ruminococcaceae*, *Desulfovibrionaceae*, *Bilophila*, *Faecalibacterium*, *Lachnospiraceae*, *Sutterella wadsworthensis*, *Faecalibacterium prausnitzii*, *Porphyromonadaceae*, *Bilophila wadsworthia*, *Parasutterella*, *Prevotellaceae*, *Parasutterella excrementihominis*, *Eubacteriaceae*, *Rikenellaceae*, *Roseburia*, *Bifidobacteriaceae*, *Alistipes*, *Clostridiaceae*, *Veillonellaceae*, *Parabacteroides*, *Barnesiella*, *Dialister*, *Acidaminococcaceae*, *Phascolarctobacterium* and *Erysipelotrichaceae.*

**Figure 4:**
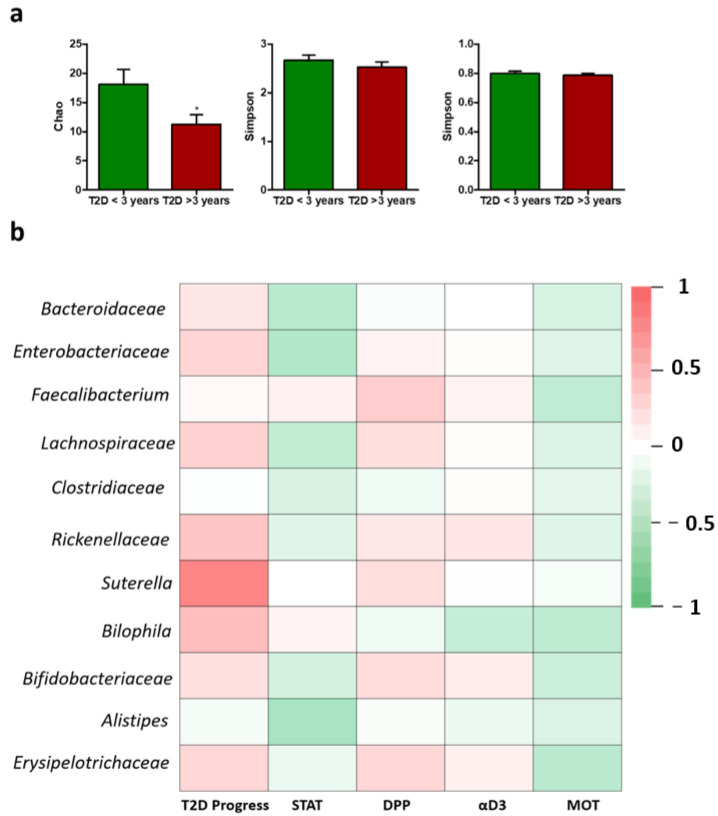
(**a**). Alpha microbiome diversity in T2D patients—Chao1, Shannon index, Simpson index. (**b**). Correlations between T2D chronicity, medication use and the top OTUs in type 2 diabetes patients. Red indicates a positive correlation, green indicates a negative correlation, while white indicates no correlation.

Next, several alpha diversity indices were calculated to analyze the microbiome differences in the samples from T2D patients. The *Chao index*, *Shannon entropy* and *Simpson* were calculated for all samples to establish the alpha diversity patterns in the cohort analyzed (Figure 4a). Chao 1, which is an estimator of phylotype richness in the analyzed samples, was significantly different between the two analyzed groups, showing that the patients having T2D for more than 2 years harbored a lower microbiome richness (* *p* < 0.05). Nevertheless, the Shannon diversity index and the Simpson index were similar among the patients analyzed.

The β diversity differences between bacterial communities found in T2D patients were calculated using the *Bray–Curtis* metric (which considers the species abundance count) and visualized by principal coordinate analysis. No significant differences were found between the two groups analyzed (Supplementary Figure S1). Correlations between the main taxa of the microbiome and the T2D progress revealed a significant correlation between *Sutterella* abundance and the likeness of developing T2D complications. Specifically, individuals having T2D for more than 3 years, who are more likely to develop complications, were enriched in *Sutterella*.

We next investigated the correlations between the main taxa identified in the microbiome of T2D individuals based on the type of medication used. Among the 50 analyzed patients, 40 were on statin treatment, 15 on domperidone (brand name Motilium), 18 on Dipeptidyl peptidase inhibitors (DPP-4-i) and 12 on αD3 supplements. DPP-4-i use was positively correlated with *Faecalibacterium* abundance, but this difference was not statistically significant. Even though *Alistipes* was negatively correlated with statin use and *Erysipelotrichaceae* were negatively correlated with domperidone use, these differences were not statistically significant (Figure 4b).

**Figure 5:**
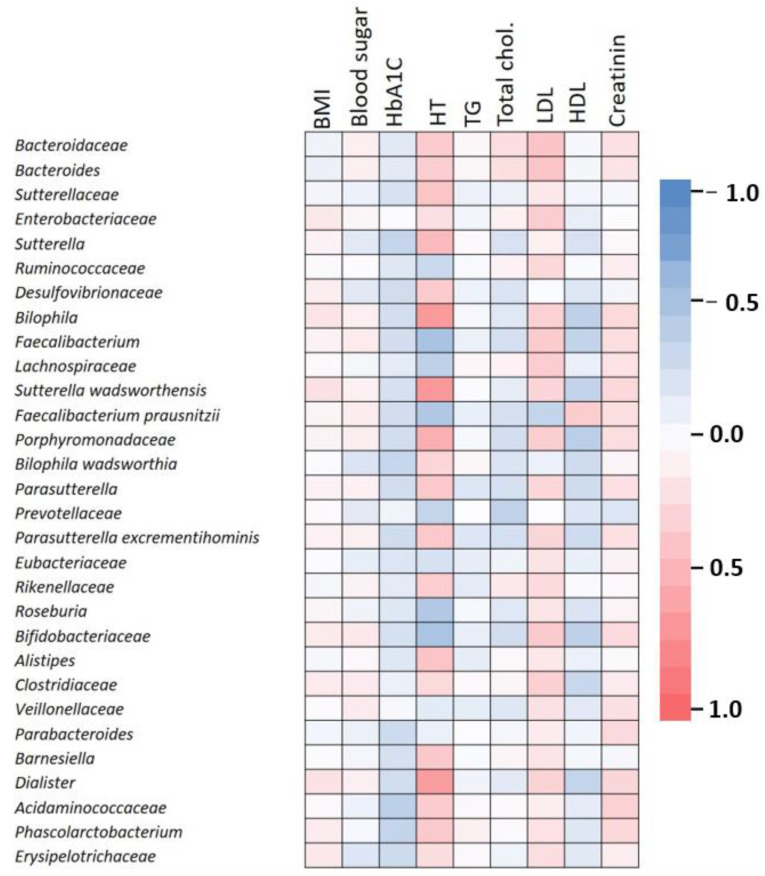
Correlations between clinical parameters and the top 30 OTUs in type 2 diabetes patients. The clinical parameters included BMI, hypertension (HT), hemoglobin A1c (HbA1c), serum cholesterol, triglycerides, HDL, LDL and creatinine. Pink indicates a positive correlation, blue indicates a negative correlation, while white indicates no correlation.

We further investigated the correlations between clinical parameters and intestinal microbiota in 50 type 2 diabetes patients. The Spearman correlation analysis was employed to investigate the interplay between different clinical values and microbial richness, in order to obtain the correlation and significant *p*-value between each clinical parameter and microbial abundance (Figure 5). The *Faecalibacterium* genus was negatively correlated with hypertension (* *p* = 0.0012) and LDL levels (* *p* = 0.0268) and positively correlated with HDL (* *p* = 0.0287). HbA1c was negatively correlated with *Phascolarctobacterium* (* *p* = 0.0352), *Acidaminococcaceae* (* *p* = 0.0165), *Bilophila wadsworthia* (* *p* = 0.0439) and *Sutterella* (* *p* = 0.0383).

Arterial hypertension was positively correlated with *Bilophila* (**** *p* < 0.0001), *Barnesiella* (* *p* = 0.0183), *Dialister* (**** *p* < 0.0001), *Acidaminococcaceae* (* *p* = 0.0267), *Phascolarctobacterium* (* *p* = 0.0186), *Alistipes* (* *p* = 0.0124), *Rikenellaceae* (* *p* = 0.0324), *Porphyromonadaceae* (*** *p* = 0.0003), *Parasutterella* (* *p* = 0.0189), *Parasutterella excrementihominis* (* *p* = 0.0186), *Sutterella* (** *p* = 0.0024), *Desulfovibrionaceae* (* *p* = 0.0258), *Bacteroidaceae* (* *p* = 0.0261) and negatively correlated with *Bifidobacteriaceae* (** *p* = 0.0015), *Roseburia* (** *p* = 0.0039), *Prevotellaceae* (* p = 0.0302) and *Faecalibacterium prausnitzii* (** *p* = 0.0031).

We also found a negative correlation between total cholesterol and *Prevotellaceae* abundance (* *p* = 0.0245). LDL levels were positively correlated with *Bacteroidaceae* (* *p* = 0.0108), *Bacteroides* (* *p* = 0.0124) and *Enterobacteriaceae* (* *p* = 0.0338).

*Dialister* was negatively correlated with hypertension (**** *p* < 0.0001) and HDL levels (* *p* = 0.0307). *Bilophila* was significantly associated with hypertension and inversely correlated with HDL cholesterol levels (* *p* = 0.0191). *Sutterella wadsworthensis* was positively linked to hypertension (**** *p* < 0.0001) and negatively with HDL (* *p* = 0.0292)

*Clostridiaceae* were positively correlated with LDL cholesterol levels (* *p* = 0.0411). Interestingly, *Bifidobacteriaceae* were positively correlated with LDL (* *p* = 0.0231) and negatively correlated with HDL cholesterol (* *p* = 0.0203). *Lachnospiraceae* were positively correlated with LDL cholesterol (* *p* = 0.0326) and negatively associated with hypertension (* *p* = 0.0203). *Faecalibacterium prausnitzii* was negatively correlated with LDL (* *p* = 0.0303) and positively correlated with HDL (* *p* = 0.0361) whereas *Porphyromonadaceae* were positively associated with LDL cholesterol (* *p* = 0.0367) and negatively associated with HDL (* *p* = 0.0139).

## 3. Discussion

In this cross-sectional study, we report significant modifications of the gut microbiome in T2D individuals. We have previously reported that patients with metabolic syndrome have a microbiome enriched in *Enterobacteriaceae* [25]. Herein, we show that the microbiome of T2D patients is significantly increased in *Enterobacteriaceae*, a family of facultatively aerobic microorganisms generally viewed as a marker of gut dysbiosis and inflammation [26]. Understanding the rationale behind the consistent enrichment of these opportunistic pathogens might provide valuable targets related to T2D pathophysiology.

Loss of butyrate producers from the gut microbiota has been constantly reported in the case of prediabetic and diabetic patients [9,21,27,28,29]. Similar to these studies, we found a significant reduction in butyrate-producing microbes such as *F. prausnitzii*, *Ruminococcus*, *Butyricicoccus* *spp.* and *Clostridium leptum* in the T2D cohort. These findings suggest an important role of butyrate producers and/or butyrate in T2D pathophysiology. Butyrate was reported to improve adiposity and glucose sensitivity [30]. High abundance of butyrate-producing microbes was characterized by improved insulin response during an oral glucose tolerance test (an indication of improved β-cell function) [31]. Hence, administration of butyrate/butyrate-producing microbes may serve as a targeted measure to prevent or delay the onset of T2D and its complications. 

Analysis of the microbially produced metabolites showed that T2D patients harbored significantly higher levels of succinate. Succinate is an organic acid produced by the microbiota and the host and can respond to tissue damage or metabolic stress. Succinate levels can build up as a consequence of dysbiosis and intestinal inflammation and can boost the colonization with potentially pathogenic microorganisms that use this organic acid as a nutrient source [32].

The main OTUs identified in T2D patients by next generation sequencing were *Bacteroides*, *Sutterellaceae*, *Enterobacteriaceae*, *Ruminococcaceae*, *Desulfovibrionaceae*, *Bilophila*, *Faecalibacterium*, *Lachnospiraceae*, *Porphyromonadaceae*, *Bilophila wadsworthia*, *Parasutterella*, *Prevotellaceae*, *Rikenellaceae*, *Roseburia*, *Bifidobacteriaceae* and *Alistipes*. These OTUs were significantly correlated with different clinical parameters including HbA1c, the lipid profile and hypertension. Microbes considered to be associated with dysbiosis such as *Bilophila* [33], *Sutterella* [34] and *Desulfovibrionaceae* [35] were correlated with a worse clinical tableau in the case of T2D patients. Conversely, the presence of *F. prausnitzii* in the T2D microbiome was associated with improved metabolic parameters such as LDL and HDL. These findings are in accordance with several studies published so far which have highlighted the beneficial effects of this butyrate-producing bacterium. Indeed, *F. prausnitzii* is negatively correlated with insulin resistance [11] and is involved in intestinal homeostasis via stimulation of mucin secretion and reduction in bacterial translocation [36]. Due to its many beneficial effects, *F. prausnitzii* has the potential to be used as a complementary or even preventive treatment for T2D and obesity-related inflammation [37]. 

High levels of LDL cholesterol and triglycerides are risk factors in the pathogenesis and development of T2D complications (i.e., diabetic nephropathy) [38,39] and we show here that certain members of the microbiota are correlated with these clinical parameters. We also report here that *Bacteroides* and *Enterobacteriaceae* are microbes significantly associated with LDL cholesterol. In line with our findings, *Bacteroides* was recently associated cholesterol and triglycerides in patients with diabetic nephropathy [40]. We show a significant correlation between the *Alistipes* and hypertension. This is in accordance with previously published data linking *Alistipes* to inflammation and epithelial changes in hypertension [41]. 

Analysis of the T2D microbiota patterns also revealed an enrichment in *Fusobacterium* and *Dialister*, taxa shown to be elevated in the gut of cancer patients [42,43]. These findings highlight the fact that dysbiosis is a culprit for both diabetes and cancer. Moreover, type 2 diabetic individuals have a higher risk of developing solid tumors including colorectal cancer [44]. Recently, the microbiota has been reported as a link between diabetes and cancer via an IL-1β and NADPH oxidase 4 dependent signaling cascade [45]. 

It is currently unknown whether associations between gut microbiota composition and T2D are different depending on the ethnic background of individuals. Here, we show that the microbiome of Romanian T2D patients is predominantly composed of *Bacteroides* species, is enriched in *Enterobacteriaceae* and depleted of beneficial butyrate-producing microbes (*Butyricoccus, F. prausnitzii*). These findings are similar to the study by Salamon et al., performed on a Polish cohort, showing that the T2D-associated microbiota was enriched in Enterobacteriaceae and low in butyrate producers such as *Roseburia* and *Faecalibacterium* [46]. The French T2D individuals also exhibited a decrease in *F. prauniztii* [11], while the US subjects harbored higher Enterobacteriaceae (*Collinsella*, and an unknown genus belonging to family *Enterobacteriaceae*) in T2D individuals [10]. Analysis of the microbiota signatures in T2D individuals from different ethnicities but living in the same geographical area (African Surinamese and South-Asian Surinamese T2D patients) also revealed a reduction in butyrate producers (such as *Anaerostipes hadrus*) [47]. The comparison in the compositional and functional features of the gut microbiota across individuals from Denmark and South India with a focus on T2D has shown that diabetics (regardless of their ethnicity) had an increased relative abundance of two OTUs from the *Lachnospiraceae* family, and a lower abundance of butyrate producers *Subdoligranulum* and *Butyricicoccus* [48]. In a Chinese–Scandinavian study sample, an increased abundance of *Escherichia* was observed in Danish and Swedish T2D study participants, but not in the Chinese T2D patients [21]. This suggests that enrichment of Enterobacteriaceae may be an effect of ethnicity or demography. *A. muciniphila* is a mucin-degrading bacterium of the phylum Verrucomicrobia reported to be associated with a healthier metabolic status in obese and overweight individuals [24,49]. Several reports indicate that *A. muciniphila* modulates glucose and lipid metabolism, as well as intestinal immunity. Moreover, metformin was reported to increase the abundance of *A. muciniphila* in the gut [50]. We have previously shown that A. muciniphila abundance is decreased in patients with metabolic syndrome [25] and we show here that this microbe is significantly reduced in the T2D-associated microbiota. Similar to our findings, *Akkermansia* abundance was shown to be decreased in Chinese T2D [51] as well as in a prediabetes Danish cohort [27].

## 4. Materials and Methods

### 4.1. Study Group

The study population (*n* = 150) was represented by 105 type 2 diabetes patients from the National Institute of Diabetes, Nutrition and Metabolic diseases N.C. Paulescu from Bucharest, Romania and 45 healthy volunteers. All the participants included in the study signed an informed consent. The clinical parameters of the study participants are listed in Table 1.

### 4.2. Microbiota Analysis

Fecal samples were collected using antiseptic handling and immediately frozen at −20 °C. DNA was extracted using the PureLink Microbiome Purification Kit (Invitrogen, Waltham, MA, USA) following the manufacturer’s instructions. DNA concentration was measured using the Qubit Broad Range kit and the Qubit 4 fluorometer (Thermo Scientific, Waltham, MA, USA). For the quantitative Real-Time PCR analysis, microbial DNA samples were diluted at a 3 ng/μL concentration. For microbiota analysis using qRT-PCR, bacterial group-specific 16S rRNA primers were used. The sequence of the primers used are listed in Table 2. Each amplification reaction included 9 ng of DNA, 2.5 nM primers and SYBR Green Master Mix (Applied Biosystems, Waltham, MA, USA). Samples were analysed on a ViiA7© Fast Real-Time instrument (Applied Biosystems, Waltham, MA, USA).

For sequencing of the microbiome, partial 16S rRNA gene sequences were amplified from extracted fecal DNA using primer pairs targeting the hypervariable regions of the 16S rRNA gene (V2-4-8 and V3-6, 7-9). The PCR products derived from amplification of specific 16S rRNA gene hypervariable regions were purified using Agencourt AMPure beads (Beckman coulter, Brea, CA, USA). Libraries were performed using the Ion Plus Fragment Library kit (Applied Biosystems) and quantified using the Ion Universal Library Quantitation kit (Cat no. A26217). Template preparation was further conducted using the ION PGM Hi-Q View OT2 kit-400. Sequencing of the amplicon libraries was performed on a 318-chip using the Ion Torrent PGM system. The individual sequence reads obtained were filtered by the Ion Reporter PGM software to discard polyclonal and low-quality reads. The sequencing data obtained was processed using Quantitative Insights Into Microbial Ecology (QIIME) [52] and to calculate downstream diversity measures, 16S rRNA Operational Taxonomic Units (OTUs) were defined at ≥97% sequence homology. All reads were classified using reference datasets (Curated Greengenes v13.5; Curated MicroSEQ(R) 16S Reference Library v2013.1).

The alpha and beta diversity graphics created in QIIME were exported from the Ion Reporter Software. In the case of alpha diversity, the Shannon curves were generated to analyze species diversity within the samples. For beta diversity analysis, cluster analysis was conducted using three-dimensional principal coordinates analysis (PCoA) with Bray–Curtis dissimilarity index and visualized in Emperor.

### 4.3. Metabolite Analysis

Sample preparation for fecal metabolome analysis was conducted as previously described [53]. Briefly, 200 mg fecal samples were suspended in 1 mL PBS, incubated at room temperature and centrifuged at 4000 rpm (1 h, 4 °C). The collected supernatant was subsequently centrifuged at 16,000 rpm (30 min, 4 °C). The supernatant obtained was filtered with a minisart-GF filter membrane (Sartorius, Göttingen, Germany) and a Whatman-25mmGD/X0 filter (Millipore, Burlington, MA, USA). Metabolite levels were quantified using commercial kits:Abbexa (Cambridge, UK) kit for butyrate measurements and Sigma Aldrich (Saint Louis, MO, USA) kits for quantification of the other metabolites.

### 4.4. Statistical Analysis

Data are shown as mean ± SEM and were graphed using GraphPad Prism 9.4.1. Differences in microbial relative abundance were tested using Unpaired t-test with Welch’s correction. A heat map based on Spearman correlations was constructed to compare the microbiota patterns and clinical parameters. The * *p* < 0.05 was considered as statistically significant. The statistical significance levels were *, *p* < 0.05; **, *p* < 0.01; ***, *p* < 0.001, **** *p* < 0.0001.

## 5. Conclusions

Our data highlight the importance of microbiome derived biomarkers in T2D and, to our knowledge, are the first reported on a significant Romanian cohort. One strength of the study is that we investigated the microbiota of a high number of middle-aged T2D patients from the Romanian population. We observed correlations between T2D gut microbiota and the host clinical parameters, which were independent of metformin treatment. Further intervention studies targeting the gut microbiota through probiotics, prebiotics, diet or other medical interventions (i.e., fecal transplant) could provide new personalized approaches in T2D treatment. Some of the limitations of the study is represented by the small sample size for the healthy control group, due to the harsh inclusion criteria (i.e., lack of comorbidities such as cardiovascular disease, etc.). Additionally, the work presented here is a cross-sectional study; hence, the observations reported here would require follow-up in a longitudinal study, as well as inclusion of populations from different ethnic backgrounds. Future research using higher-resolution shotgun sequencing is needed to clarify the role of ethnicity in the association between T2D and gut microbiota composition.

## Figures and Tables

**Table 1:** Characteristics of Study Participants.

Characteristic	HC	T2D
Age	57 ± 10.30	63 ± 12.25
Sex (F/M)	30/15	74/31
BMI	24.9 ± 2.115	30 ± 4.39
Blood pressure (mmHg): systolic	110 ± 2.20	139.5 ± 2.9
Blood pressure (mmHg): diastolic	62 ± 1.89	87.5 ± 1.6
HbA1c (%)	5.4 ± 0.29	6.5 ± 0.3
HDL	65 ± 4.89	48 ± 7.52
LDL	98 ± 16.89	116 ± 31.45
TG	89 ± 18.97	129 ± 53.67
Statin (number/total)	9/45	92/105
Metformin (number/total)	n/a	65/105

**Table 2:** The sequences of the primers used.

Taxonomic Target	Sequence
*Actinobacteria*	tgtagcggtggaatgcgc aattaagccacatgctccgct
*Verrucomicrobia*	tcaggtcagtatggcccttat cagttttcaggatttcctccgcc
*Bacteroides spp*.	cctacgatggataggggtt cacgctacttggctggttcag
*Butyricicoccus* *spp.*	acctgaagaataagctcc gataacgcttgctccctacgt
*Betaproteobacteria*	aacgcgaaaaaccttacctacc tgccctttcgtagcaactagtg
*Gamma proteobacteria*	gctaacgcattaagtaccccg gccatgcagcacctgtct
*Akkermansia muciniphila*	gcg tag gct gtt tcg taa gtc gtg tgt gaa ag gag tgt tcc cga tat cta cgc att tca
*Eubacteria*	act cct acg gga ggc agc agt att acc gcg gct gct ggc
*Lactobacillus* *spp.*	acg agt agg gaa atc ttc ca cac cgc tac aca tgg ag
*BPP*	ggtgtcggcttaagtgccat cggacgtaagggccgtgc
*Clostridium leptum*	gcacaagcagtggagt cttcctccgttttgtcaa
*Ruminococcus* *spp.*	actgagaggttgaacggcca cctttacacccagtaattccgga
*Fusobacterium* *spp.*	acctaagggagaaacagaacca cctgcctttaattcatctccat
*Firmicutes*	ggagcatgtggtttaattcgaagca agctgacgacaaccatgcac
*Bacteroidetes*	ggaacatgtggtttaattcgatgat agctgacgacaaccatgcag
*F. prausnitzii*	cccttcagtgccgcagt gtcgcaggatgtcaagac

## Data Availability

The data presented in this study are available on request from the corresponding author. The data are not publicly available due to privacy/ethical restrictions.

## Supplementary Materials

The supporting information can be downloaded at: https://www.mdpi.com/1422-0067/23/23/15023/s1.
