# Supplementary material for: Snapshot into the Type-2-Diabetes-Associated Microbiome of a Romanian Cohort
Source: Int J Mol Sci. 2022 Nov 30;23(23):15023. doi: 10.3390/ijms232315023 (PMC9741184; doi:10.3390/ijms232315023)
Supplement: Supplementary file 1 [file ijms-23-15023-s001.zip › ijms-2032732-supplementary.pdf]

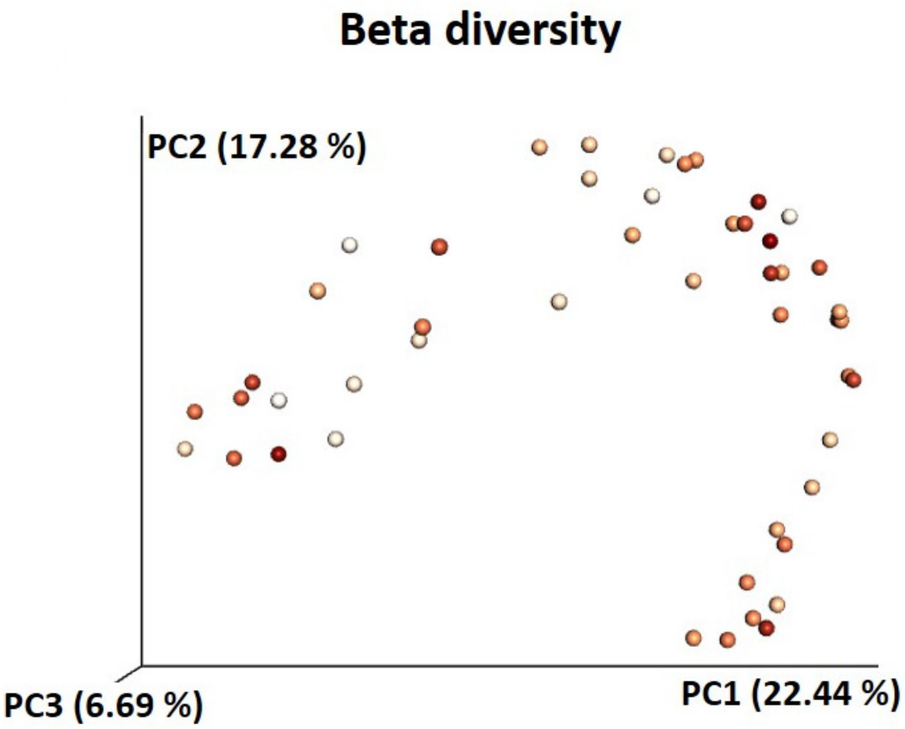

Supplementary Figure S1. Beta diversity analysis at species level: principal coordinate analysis (PCoA) based on Bray–Curtis dissimilarity (species level)
